# Phenolic water toxins: redox mechanism and method of their detection in water and wastewater†

**DOI:** 10.1039/d1ra05669g

**Published:** 2021-11-05

**Authors:** Tayyaba Kokab, Afzal Shah, Jan Nisar, Muhammad Naeem Ashiq, M. Abdullah Khan, Sher Bahadar Khan, Esraa M. Bakhsh

**Affiliations:** Department of Chemistry, Quaid-i-Azam University Islamabad 45320 Pakistan afzals_qau@yahoo.com; National Centre of Excellence in Physical Chemistry, University of Peshawar Peshawar 25120 Pakistan; Institute of Chemical Sciences, Bahauddin Zakaryia University Multan 6100 Pakistan; Renewable Energy Advancement Laboratory, Department of Environmental Sciences, Quaid-i-Azam University Islamabad 45320 Pakistan; Department of Chemistry, King Abdulaziz University P.O. Box 80203 Jeddah 21589 Saudi Arabia

## Abstract

Phenolic pollutants are highly toxic and persistent in the environment. Their efficient detection is a pressing social demand. In this regard we introduce a novel ultrasensitive electroanalytical platform for the individual and synchronized detection of three phenolic isomers commonly known as hydroquinone (HQ), resorcinol (RC), and catechol (CC). The sensing device consists of a glassy carbon electrode (GCE) modified with functionalized carbon nanotubes (fCNTs) and gold–silver (Au–Ag NPs) bimetallic nanoparticles. The sandwiched scaffold represented as fCNTs/Au–Ag NPs/fCNTs/GCE efficiently senses HQ, RC, and CC with detection limits of 28.6 fM, 36.5 fM and 42.8 fM respectively. The designed sensor is more promising than reported sensors for phenolic toxins in the context of high sensitivity, selectivity, and rapid responsiveness. The designed sensor also shows the qualities of stability, reproducibility, reliability, and selective recognition capacity for target analytes in multiple real water samples. Moreover, computational calculations explain the function of the electrode modifier in facilitating charge transfer between the transducer and analytes.

## Abstract 2

Phenolic pollutants are highly toxic and persistent in the environment.

## Graphical abstract

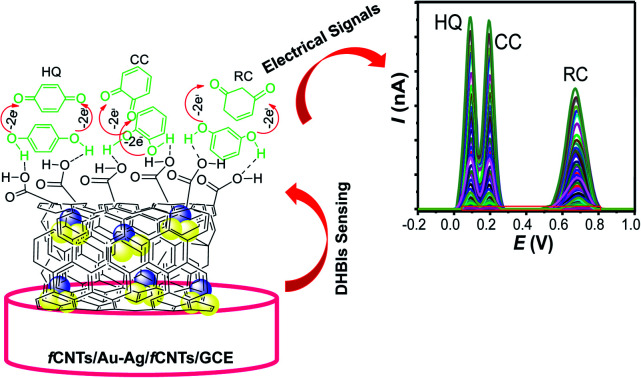

## 1. Introduction

Hydroquinone (HQ), resorcinol (RC), and catechol (CC) are extensively used in tanning, metallurgy, petrochemicals, and cosmetics.^1–4^ Ingestion of these isomeric compounds even at a miniscule level is detrimental owing to their genotoxicity, carcinogenicity and poor degradability.^5^ A literature survey revealed the involvement of HQ, CC, and RC in causing headaches, fatigue, acute myeloid leukemia, gastrointestinal tract degeneration, liver malfunction, hemolysis, and DNA damage.^6^ The US Environmental Protection Agency (EPA) and World Health Organization (WHO) have proclaimed these chemicals as primary toxins.^7^ Hence, it is a social obligation to develop a protocol for the concurrent minute level detection of HQ, CC, and RC in environmental samples.

Although a considerable progress for the detection of individual dihydroxybenzene isomers (DHBIs) *i.e.*, HQ, CC, and RC has been made, but simultaneous detection of minute level concentration of these isomers is still a challenging task. To deal with this challenge a number of reports on synchronized micro to nanomolar detection of HQ, RC, and CC are available,^2–6,8–15^ however, new materials are urgently required for picomolar to femtomolar simultaneous sensing of these isomeric water toxins. In this regard carbon nanotubes (CNTs) are promising materials for electrode modification by virtue of their structure that supports efficient electron transport.^16–21^ The conducting features of CNTs are further improved by the incorporation of metal nanoparticles (NPs).^22–25^ Biocompatible nanomaterials such as metal (iron, gold, zinc, copper and silver *etc.*) nanoparticles (M NPs) bestow unique electrochemical recognition capacity to the electrode for organic analytes.^17,18^ Precious metal nanoparticles (NPs) in conjunction with CNTs confer the electrode surface with electroactive features.^21^ Several authors have demonstrated that noble bimetallic alloy nanoparticles owe exceptional affinity for organic analytes than their single-metal analogues.^23,24^ Electrochemical results of Au–Ag NPs deposited on GCE reveal that Au–Ag NPs (2 : 1) are active for the distinct electrocatalytic oxidation of target isomers. Considering the remarkable electrocatalytic and biocompatibility characteristics, robust adsorption ability and excellent conducting features of noble bimetallic NPs,^22–25^ we integrated CNTs with gold-silver NPs for the development of a highly sensitive nanosensor.

The recognition capacity of the sensing surface was further enhanced by the induction of the Au–Ag NPs between the layers of –COOH functionalized carbon nanotubes CNTs (COOH-fCNTs) to form a sandwiched nanocomposite (fCNTs/Au–Ag NPs/fCNTs). The functional groups (–OH, –COOH) of fCNTs offer electrostatic affinity for Au–Ag NPs which in turns prohibit their agglomeration.^26,27^ Consequently, the distribution of bimetallic NPs onto large active surface of fCNTs network improves the physiochemical features such as conductivity, signal amplification and electrocatalytic performance of the nanosensor for the discrimination of target isomeric analytes. Literature survey^2–6,8–15^ reveals that the reported sensors for the solo and concurrent detection of DHBIs demand improvement in sensitivity. Our designed sensing platform based on Au–Ag NPs decorated fCNTs present practically superior figures of merit for the target phenolic isomers than a plethora of electrode modifiers such as protein units (amino acids), organic compounds (2,5-dimercapto-1,3,4-thiadiazole, 3,5-diamino-1,2,4-triazole-CO covalent organic frame work film, polydiallyldimethylammonium chloride), inorganic conductors (metal NPs, amino functionalized ordered mesoporous silica), and carbonaceous compounds (nitrogen-doped carbon nanofibers, porous graphene, graphene sheets embedded carbon).^2–15^ To the best of our knowledge, this is the first report about a sensor development that selectively senses the target isomers concurrently with detection limits in the femtomolar range.

## 2. Experimental

### 2.1. Chemicals

Chemicals of analytical grade procured from Alfa Aesar, Merck Germany, or/and Sigma Aldrich were used in this work. Solutions of HCl, H_2_SO_4_, H_3_BO_3,_ NaOH, KCl, C_2_H_3_NaO_2_, CH_3_COOH, H_3_PO_4_, and Na_3_PO_4_ were used for the preparation of different supporting electrolytes. Doubly deionized water (ddw) was used to prepare analyte's solutions, phosphate buffer solution (PBS), Britton–Robinson buffer (BRB), acetate buffer solution (ABS), and solutions of interfering agents. Moreover, the glass kits were used after cleaning with conc. HNO_3_ and double distilled water to avoid chances of contamination.

### 2.2. Synthesis of bimetallic nanoparticles and functionalization of CNTs

**Fig. 1:**
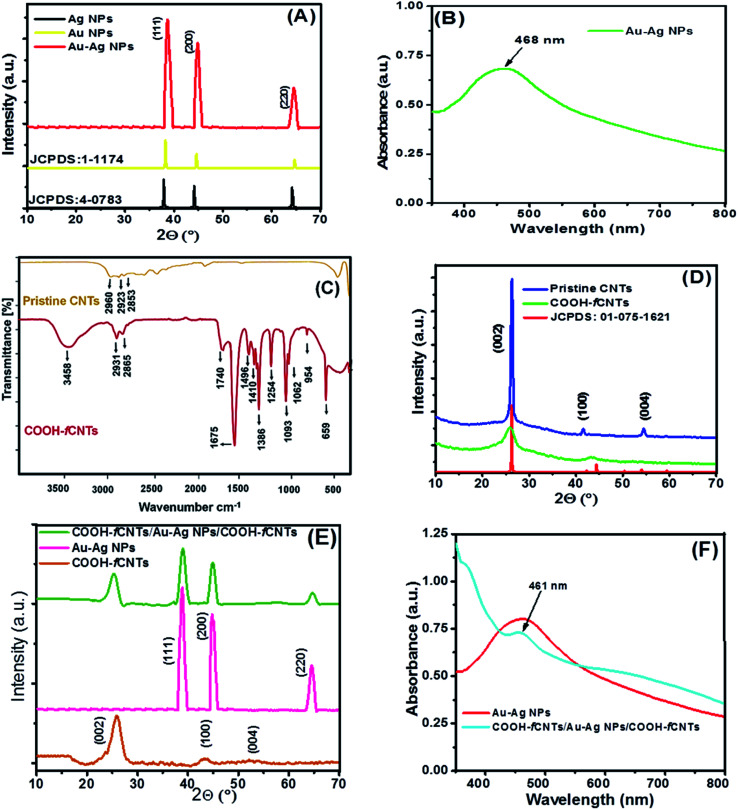
Structural characterization: (A) The XRD of Au–Ag NPs (B) UV-Vis spectrum of Au–Ag NPs (C) FTIR spectrum of pristine and functionalized CNT (D) XRD pattern of pristine and functionalized CNT (E) XRD pattern of COOH-fCNTs/Au–Ag NPs/COOH-fCNTs, Au–Ag NPs and COOH-fCNTs (F) UV-Visible spectra of COOH-fCNTs/Au–Ag NPs/COOH-fCNTs and Au–Ag NPs.

Au–Ag NPs (2 : 1) were prepared *via* our reported co-precipitation procedure.^28^ The growth of the synthesized NPs was confirmed by UV-Vis spectroscopy and XRD analysis as shown in Fig. 1A and B. The NPs were obtained by centrifugation subsequently purified with acetone and ethanol. The Au–Ag NPs (2 : 1) 1 mg mL^−1^ dispersion was made in ethanol by ultrasonic agitation for 30 minutes. Commercially procured pristine CNTs were refined and functionalized under harsh oxidative conditions. For this purpose, 0.2 L of 1 : 3 v/v of 2 M HNO_3_ and 2 M H_2_SO_4_ solution was added with 1 g CNTs. The resulting solution was refluxed at 140 °C for 2 h to speed up the oxidation process. Then, the mixture was separated by 0.25 μm cellulose membrane and the precipitates were washed continuously with ddw until neutral pH of the filtrate. The precipitates were dehydrated at 100 °C for 3 hours in a curing oven and the resultant product designated as COOH-fCNTs was characterized *via* FTIR and XRD as shown in Fig. 1C and D.^29^

### 2.3. Modification of GCE with fCNTs/Au–Ag NPs/fCNTs

Before modification physiochemical cleaning steps were performed to upgrade the O/C ratio of the GCE surface.^30^ The electrode surface was first gently scrubbed on a nylon cushion with γ-Al_2_O_3_ paste until the appearance of a polished surface. It was then rinsed with ddw, ethanol, and aq. HNO_3_ (1 : 1, v/v) followed by drying with a drier at room temperature. Afterwards, the chemical cleaning of GCE was performed in the potential window of −0.2 V to + 1.8 V, *vs.* Ag/AgCl (3 M KCl) reference electrode, to obtain multiple reversible cyclic voltammograms in 0.1 M HClO_4_ until the attainment of a reproducible surface.

The GCE fabrication was done by drop coating of fCNTs and Au–Ag NPs *via* layer-by-layer (LBL) and mixing methods.^31^ For LBL, a 4 μL droplet of COOH-fCNTs (1 mg mL^−1^) black suspension was carefully casted on a pre-cleaned GCE surface and dried in vacuum oven. Then, 1 μL droplet of Au–Ag NPs (1 mg mL^−1^) was coated on the already prepared fCNTs/GCE. After that, a 4 μL droplet of COOH-fCNTs solution was placed and dried on the Au–Ag NPs/fCNTs/GCE to develop fCNTs/Au–Ag NPs/fCNTs/GCE. While in the mixing method of electrode modification, one mg of Au–Ag NPs was added in 5 mL of 1 mg mL^−1^ fCNTs dispersion and mixed under ultra-sonication for 30 min. A 5 μL droplet of the prepared mixture was casted and dried on the GCE surface to prepare Au–Ag NPs/fCNTs/GCE. For comparison purpose, Ag NPs/GCE, Au–Ag (1 : 1)/GCE, Au NPs/GCE, Au–Ag (2 : 1)/GCE, Au–Ag (1 : 2)/GCE, Au–Cu (1 : 1)/GCE, Au–Cu (1 : 2)/GCE, Au–Cu (1 : 3)/GCE, Au–Cu (3 : 1)/GCE, Au–Cu (2 : 1)/GCE, Ag–Cu (1 : 1)/GCE, COOH-fCNTs/GCE, pristine CNTs/GCE, and NH_2_-fCNTs/GCE were also devised. Before use all the fabricated GCEs were thoroughly rinsed with ddws and PBS to strip off any loosely attached modifier molecules.^16^ Fabrication steps for the preparation of fCNTs/Au–Ag NPs/fCNTs/GCE can be seen in Scheme S1.†

### 2.4. Instruments for characterization and design of experiment

The synthesized nanomaterials, crystallographic configuration and structural properties were analysed at XRD (model # Panalytical 3040/60 X pert PRO diffractometer) having beam source CuKα at 40 kV/40 mA voltage/current. The spectrum was obtained at a scan rate of 0.01° s^−1^ from 10° to 70° (2*θ*). Likewise, the optical behaviour of the synthesized materials was studied at UV-Visible spectrophotometer (model # Shimadzu 1601) in the wavelength range of 200–1100 nm and path length of 1 cm. Further, the functionalization of the as-synthesized products was confirmed through Fourier transform infrared spectroscopy (model # Thermo Scientific Nicolet 6700 FTIR spectrometer) at 400–4000 cm^−1^ wavelength region. Moreover, the chemical composition and morphological features of the as-synthesized products were probed through scanning electron microscopy (SEM) and energy-dispersive X-ray spectroscopy (EDX) (model # ZEISS EVO 40 Merlin, Carl Zeiss). Electrochemical characterization was performed on Metrohm Auto lab PGSTAT302N Switzerland. A 3-electrode convectional system was used which consisted of working electrode bare and modified glassy carbon electrode; Ag/AgCl (conc. KCl) reference electrode; and counter electrode (CE) made of platinum wire. All electrochemical experiments were operated under an inert environment.

### 2.5. Real water samples

To test the viability of the designed fCNTs/Au–Ag NPs/fCNTs/GCE sensor for the synchronized evaluation of the HQ, CC, and RC, multiple real-time water samples consisting of drinking, tap, spring, rain, lake, river, and sea water samples were used. The target analytes were also investigated in complex matrixes such as artificial wastewater and vegetable juices (spinach and onion juice). Before use all water samples were filtered several times by Whatman filter paper of pore size 8 μm to eliminate impurities and diluted with 1 : 1 PBS buffer of pH 6. Artificial wastewater samples containing 5 μM industrial pollutants such as metal ions, organic toxins other than the target analytes, surfactants *etc.* were also prepared. Moreover, before SWASV analysis, onion and spinach samples were crushed into juices, then centrifuged for 15 min at 5000 RPM and the resultant supernatants were diluted twenty times with ddw. After voltammetric analysis of all the real samples, % recoveries of the stated isomers were determined by a usual spiking method to authenticate the precision and anti-interferent ability of the proposed procedure.

### 2.6. Computational calculations

Theoretical studies of the phenolic isomers and their intermolecular interactions with the designed fCNTs/Au–Ag NPs/fCNTs nanocomposite were performed by DFT, and M06-2X calculations on Gaussian 09 software to find relationship between chemical descriptors and experimental data. Molecular geometries of the isomers and their oxidized products were optimized by using DFT (B3LYP) with basis set 6-311G++ (d, p) and the obtained data were used for calculations of the quantum molecular descriptors.^32^ Then, the catalytic role of the sensor was probed by M06-2X functional that indirectly accounts the dispersion interactions (hydrogen bonding and van der Waals forces) of sensor/dihydroxybenzene isomers (DHBIs) in aqueous media. Firstly, the geometries of Au–Ag NPs (triatomic Au(2)–Ag(1) system) and COOH-fCNTs (4 COOH groups on armchair (5, 5)) were optimized through function M06-2X with LaNL2DZ, and 6-311G++ (d, p) basis sets respectively and then their quantum features were estimated. Afterwards, the optimization of the blended systems (optimized DHBIs on the Au–Ag NPs surface) was carried out at function M06-2X and basis set LANL2DZ and the quantum descriptors were calculated for their reactivities and binding energies. Similarly, the optimization of the merged systems (optimized DHBIs at Au–Ag NPs/COOH-fCNTs surface) was performed at function M06-2X and basis set LANL2DZ followed by computing their reactivities and binding energies. Notably, the surfaces on which DHBIs molecules configured parallel to Au–Ag NPs/COOH-fCNTs were processed to maximize the π–π stacking interactions in CNTs/DHBIs structures. From the data, the binding energies Δ*E* (eV) were calculated by the following equations;Δ*E* = *E*_(Au-Ag NPs-DHBIs)_ − (*E*_Au-Ag NPs_ + *E*_DHBIs_)Δ*E* = *E*_(fCNTs-Au-Ag NPs-DHBIs)_ − (*E*_fCNTs_ + *E*_Au-Ag NPs_ + *E*_DHBIs_)where, *E*_(Au-Ag NPs-DHBIs)_ and *E*_(fCNTs-Au-Ag NPs-DHBIs)_ are the total interaction energies of the blended systems while *E*_fCNTs_, *E*_Au-Ag NPs_, and *E*_DHBIs_ are the energy values of the single structures.^33^

## 3. Results and discussion

The oxidation response of dihydroxybenzene isomers (DHBIs) was investigated at Ag NPs/GCE, Au NPs/GCE, Au–Ag (1 : 1)/GCE, Au–Ag (2 : 1)/GCE, Au–Ag (1 : 2)/GCE, Au–Cu (1 : 1)/GCE, Au–Cu (1 : 2)/GCE, Au–Cu (1 : 3)/GCE, Au–Cu(2 : 1)/GCE, Au–Cu (3 : 1)/GCE, and Ag–Cu (1 : 1)/GCE, pristine CNTs/GCE, NH_2_-fCNTs/GCE, COOH-fCNTs/GCE and COOH-fCNTs/Au–Ag (2 : 1) NPs/COOH-fCNTs/GCE. The best response was obtained using COOH-fCNTs/Au–Ag (2 : 1) NPs/COOH-fCNTs as the recognition layer of the nanosensor. Characterization details and electrochemical performance of the designed nanosensor for DBHIs analysis have been presented in the following sections.

### 3.1. Structural description of the nanocomposite

The synthesis of bimetallic Au–Ag NPs, functionalization of CNTs, and development of fCNTs/Au–Ag NPs/fCNTs nanocomposites were verified by XRD, UV-Vis spectroscopy and FTIR analysis. The XRD pattern of Au–Ag NPs demonstrated in Fig. 1A has three distinct diffraction peaks at 38.7°, 44.5° and 64.3° which are indexed as (111), (200), and (220) crystallographic planes of the face centered cubic (FCC) structure. The crystalline nature of Au–Ag NPs corresponds to JCPDS cards 00-001-1174 & 00-004-0783. Owing to analogous lattice constants of Au and Ag metals, their *d* spacing and 2*θ* values are very close to each other. This makes precise assignment of individual reflections of bimetallic Au–Ag NPs virtually difficult. The relatively broad high intensity diffraction peaks of Au–Ag bimetallic NPs encompassing 2*θ* positions of monometallic peaks suggest the development of Au–Ag alloy structure. The mean size of Au–Ag NPs was calculated as ∼32 nm. In addition, the presence of characteristic (002) and (100) planes at 25.8° and 44.1° ensures the crystalline graphitic CNTs (Fig. 1D). The decrease in intensities and broadening of the peaks corresponding to loss of crystallinity in COOH-fCNTs pattern compared to pristine CNTs confirm functionalization that leads to disruption of the CNTs' wall.^29^ Whereas, all individual peaks with a small loss in intensities and slight broadening are present in the XRD pattern of the composite NPs/COOH-fCNTs. Fig. 1E shows the XRD pattern of COOH-fCNT*s*/Au–Ag NPs/COOH-fCNTs nanocomposite that confirms the presence of the FCC nature of Au–Ag NPs decorated onto the COOH-fCNTs matrix. The diffraction peak at 25.3° corresponds to COOH-fCNTs while other diffraction peaks at 39°, 44.9° and 64.8° can be assigned to the presence of FCC Au–Ag NPs in the nanocomposite. The XRD pattern endorses the synthesis of COOH-fCNTs/Au–Ag NPs/COOH-fCNTs nanocomposite. Moreover, investigation of the optical behaviour of Au–Ag NPs using UV-visible spectroscopy demonstrates surface plasmon resonance (SPR) range centred at 468 nm (Fig. 1B and F). This signal of Au–Ag alloy appearing between the characteristic SPR bands of Au and Ag NPs further validates the creation of Au–Ag alloy NPs.^28^ FTIR analysis shows the representative peaks of pristine and fCNTs. The signal matching to the C–H elongating vibration of methylene at 2900–2850 cm^−1^, broad –OH band in –COOH functionalized fCNTs at 3458 cm^−1^ and the peak of carbonyl (–C=O) group of carboxylic acid at 1740 cm^−1^ (Fig. 1C) confirms the successful functionalization of pristine CNTs by HNO_3_ and H_2_SO_4_ acids treatment. The peaks within 1675 cm^−1^ to 1496 cm^−1^ can be related to different acetyl/ketone/quinone (–C=O) groups and the C–C vibrations of benzene rings. The bands in the range of 1450 cm^−1^ to 650 cm^−1^ corresponding to C–H deformation (1439 cm^−1^), COO-stretch (1410 cm^−1^), C–O deformation (1386 cm^−1^), C–H symmetric stretching (1254 cm^−1^), C–O stretching vibrations (1151 cm^−1^), C–O–C vibrations (1093 cm^−1^), C–H in-plane deformation (1062 cm^−1^), C–C stretching (954 cm^−1^) and O–C=O bending (659 cm^−1^) infers effective acidification of CNTs.^29^

### 3.2. Morphological characterization of designed sensor

**Fig. 2:**
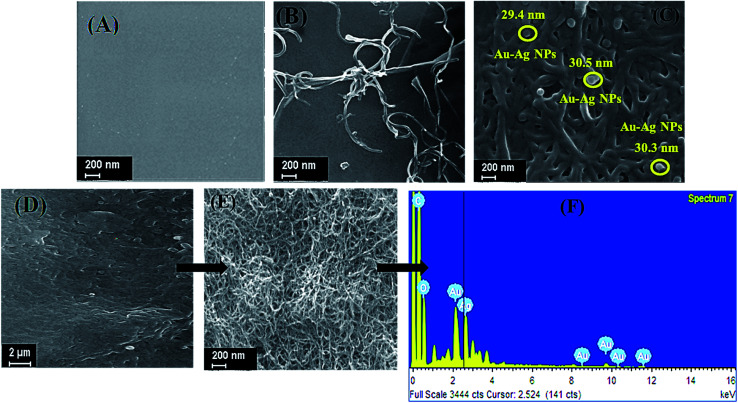
The SEM images of (A) bare GCE, (B) fCNTs fabricated GCE, (C) Au–Ag NPs/fCNTs/GCE, (D) fCNTs/Au–Ag NPs/fCNTs/GCE and (E) EDX spectra of fCNTs/Au–Ag NPs/fCNTs/GCE (F).

The morphology of layer by layer (LBL) modified electrochemical nanosensor was examined by SEM coupled with EDX. Fig. 2 illustrates the SEM of bare GCE, fCNTs/GCE, Au–Ag NPs/fCNTs/GCE, and fCNTs/Au–Ag NPs/fCNTs nanocomposite modified GCE. Physiochemically cleaned bare GCE has a smooth surface (Fig. 2A) while threads of fCNTs are observable in fCNTs/GCE (Fig. 2B). The surface structure and surface area of fCNTs/GCE are noticeably altered by the addition of Au–Ag NPs (Fig. 2C). Spherical Au–Ag NPs are homogeneously dispersed at the densely packed tubular profile of fCNTs. The average size of ∼30 nm of the Au–Ag NPs validates the size estimated from their XRD (Fig. 1A). A complete pattern of fCNTs/Au–Ag NPs/fCNTs/GCE presents a porous microstructure (Fig. 2D) as required for effective absorption of the analytes. EDX analysis of the fCNTs/Au–Ag NPs/fCNTs confirms the carbon, oxygen, gold, and silver elements presence (Fig. 2E). Moreover, the EDX spectrum Au/Ag ratio (2 : 1) verifies successful synthesis of the nanospheres.

### 3.3. Electrochemical characterization of the designed nanosensor

The designed nanosensor was first characterized by CV and EIS techniques. The key performance metrics about electron transduction mechanism, active surface area, extent of immobilization of the electrode modifier, and impedance variation at the bare and modified electrodes surfaces were obtained according to the reported method.^34^ The noticeable amplified and shifted reversible signals of the standard redox reporter (K_3_[Fe(CN)_6_]) at fCNTs/Au–Ag NPs/fCNTs/GCE propose exceptional electrocatalytic behavior of the nanocomposite that facilitates faster charge transport between the analyte and transducer (Fig. S1A†). An observation of Table S1† reveals that the working surface area of the electrodes as determined by Randles–Sevcik equation^2^ are 0.02 cm^2^ (bare GCE), 0.04 cm^2^ (Au–Ag NPs GCE), 0.07 cm^2^ (Au–Ag NPs/fCNTs/GCE) and 0.11 cm^2^ (fCNTs/Au–Ag NPs/fCNTs/GCE) respectively. Likewise, EIS analysis was performed and the corresponding Nyquist plots for the data obtained at bare GCE, Au–Ag NPs/GCE, Au–Ag NPs/fCNTs/GCE and fCNTs/Au–Ag NPs/fCNTs/GCE can be seen in Fig. S1B and Table S1.† The significantly smaller charge transfer resistance *R*_ct_ (1.5 × 10^−5^ Ω) of the nanosensor as compared to *R*_ct_ (6450 Ω) at bare GCE points to improved charge transduction through the modified electrode represented as fCNTs/Au–Ag NPs/fCNTs/GCE. EIS results also validate immobilization of the recognition layer of fCNTs/Au–Ag NPs/fCNTs at the GCE with a uniform surface (supported from CPE and *n* values).^34–36^

### 3.4. Voltammetric analysis for examining the nanosensor performance

**Fig. 3:**
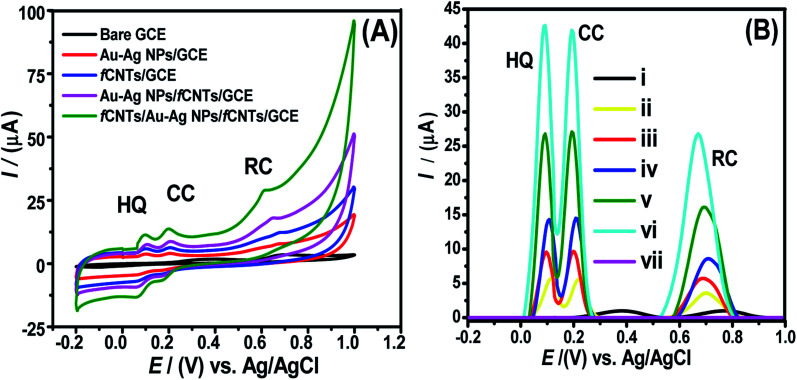
(A) Cyclic voltammograms of HQ (12.5 μM), CC (15 μM), and RC (17.5 μM) mixture at bare GCE, Au–Ag NPs/GCE, Au–Ag NPs/fCNTs/GCE, fCNTs/GC electrode and fCNTs/Au–Ag NPs/fCNTs/GCE in PBS of pH 6 at a sweep rate of 100 mV s^−1^. (B) The SWASV of HQ (10 μM), CC (12.5 μM), and RC (15 μM) mixture attained at (i) bare GCE (ii) Au–Ag NPs/GCE (iii) CNTs/GCE (d) fCNTs/GCE (iv) Au–Ag NPs/fCNTs/GCE, and (v) fCNTs/Au–Ag NPs/fCNTs/GCE (vi) fCNTs/Au–Ag NPs/fCNTs/GCE in 1 : 1 aq. solvent and PBS (pH 6) stripping solvent at a sweep rate 100 mV s^−1^ keeping deposition potential of 0 V and accumulation time of 200 s.

Cyclic and square wave anodic stripping voltammetric analysis of the HQ, RC, and CC mixture were performed at the bare and modified GCEs. At bare GCE, the HQ-CC overlapped oxidation band came to sight around 378 mV while the oxidation signal of RC emerged at 695 mV. In contrast, well-distinguished signals of the three DHBIs appeared at all modified GCEs with significantly higher currents at the fCNTs/Au–Ag NPs/fCNTs/GCE (Fig. 3A). Both the Ipc and Ipa peaks of HQ and CC appeared at fCNTs/Au–Ag NPs/fCNTs/GCE. The smaller reduction peak currents than the oxidation signals suggests quasireversible processes that involve charge transfer reaction after that chemical reaction (EC mechanism).^5^ However, the cyclic voltammograms display a small oxidation peak current of RC at 649 mV with no corresponding reduction peak suggesting the instability of the oxidized product of RC at the designed sensor surface. In addition, the SW voltammograms of the DHBIs show a more clear difference in the response at the bare and modified GCEs (Fig. 3B). The signals of the DHBIs are fused resulting in a broad wave at the bare GCE while well resolved signals are observable at the modified nanosensors. Moreover, a significant shift in the signals of DHBIs at fCNTs/Au–Ag NPs/fCNTs/GCE than the other modified GCEs endorses its highest electrocatalytic activity. The gradual decrease of overpotential from RC to CC to HQ can be related to the increase in their electronic densities that lead to increase in electroactivity of these isomers.^14,37^ The shifting of peaks of HQ (92 mV), CC (192 mV), and RC (671 mV) to low positive potentials at the fCNTs/Au–Ag NPs/fCNTs/GCE suggests the absence of CC interference in HQ oxidation which excludes the fouling of the electrode surface by their oxidized products. Interestingly the difference of peak potentials Δ*E*_p(HQ-CC)_ (100 mV) and Δ*E*_p(CC-RC)_ (479 mV) is large enough to ensure synchronized sensing of HQ, CC, and RC at the designed fCNTs/Au–Ag NPs/fCNTs/GCE. Hence, SWASV was applied for the simultaneous sensing of the three isomers.

### 3.5. Optimization of conditions and working principle of the nanosensor

**Fig. 4:**
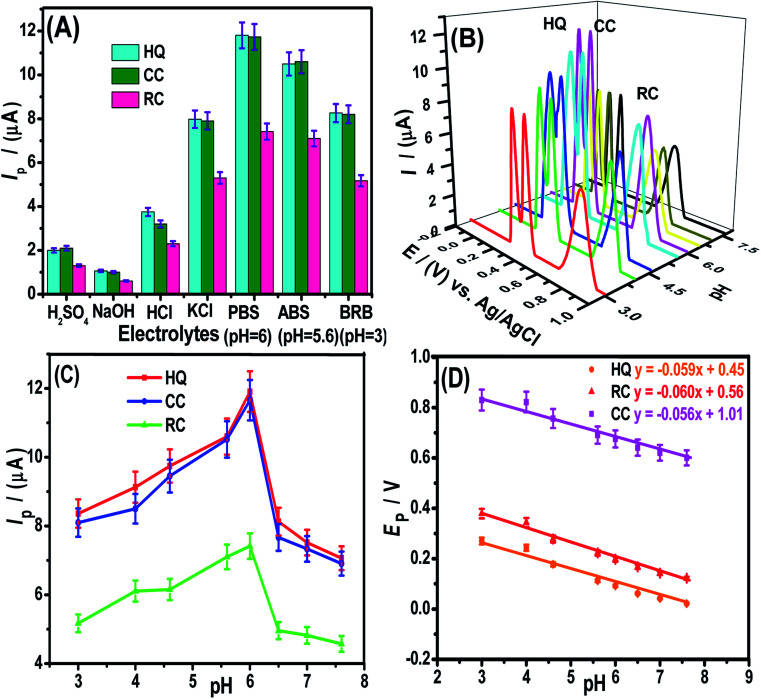
(A) Bar graph showing the influence of various supporting media such as 0.1 M H_2_SO_4_, 0.1 M NaOH, 0.1 M HCl, 0.1 M KCl, PBS (pH 6), ABS (pH 5.6), and BRB (pH 3) on the SWASV peak currents of HQ (10 μM), CC (12.5 μM), and RC (15 μM) mixture at deposition potential of 0 V and deposition time of 5 s at 100 mV s^−1^ scan rate using LBL modified GCE with 4 μL fCNTs/1 μL Au–Ag NPs/4 μL fCNTs. (B) The SWASVs as a function of pH obtained under the same conditions (C) plots of *I*_p_ of HQ, CC, and RC *vs.* pH (D) plots of *E*_p_ of HQ, CC, and RC *vs.* pH along with regression equations.

The modifier amount and pre-concentration (deposition potential, and time) step were tested to get optimized conditions for intense signals of DHBIs (Fig. S2–S3†). The scan rate effect (Fig. S4†) and influence of the pH of the medium (Fig. 4) were also studied for probing the redox mechanism of DHBIs at the fCNTs/ZnO/fCNTs/GCE surface. The nature of stripping electrolytes and their pH values critically affect the redox signals of analytes and their deposition process at the sensor surface. In this regard, a series of acidic, basic, neutral, and buffered stripping electrolytes mentioned in Fig. 4A were studied for HQ (10 μM), CC (12.5 μM) and RC (15 μM) analysis at the fCNTs/Au–Ag NPs/fCNTs/GCE. Intense and reproducible signals of the isomers were observed in a solution buffered with PBS (pH 6). Therefore, it was chosen as the working medium for the synchronized analysis of the DHBIs.

**Scheme 1:**
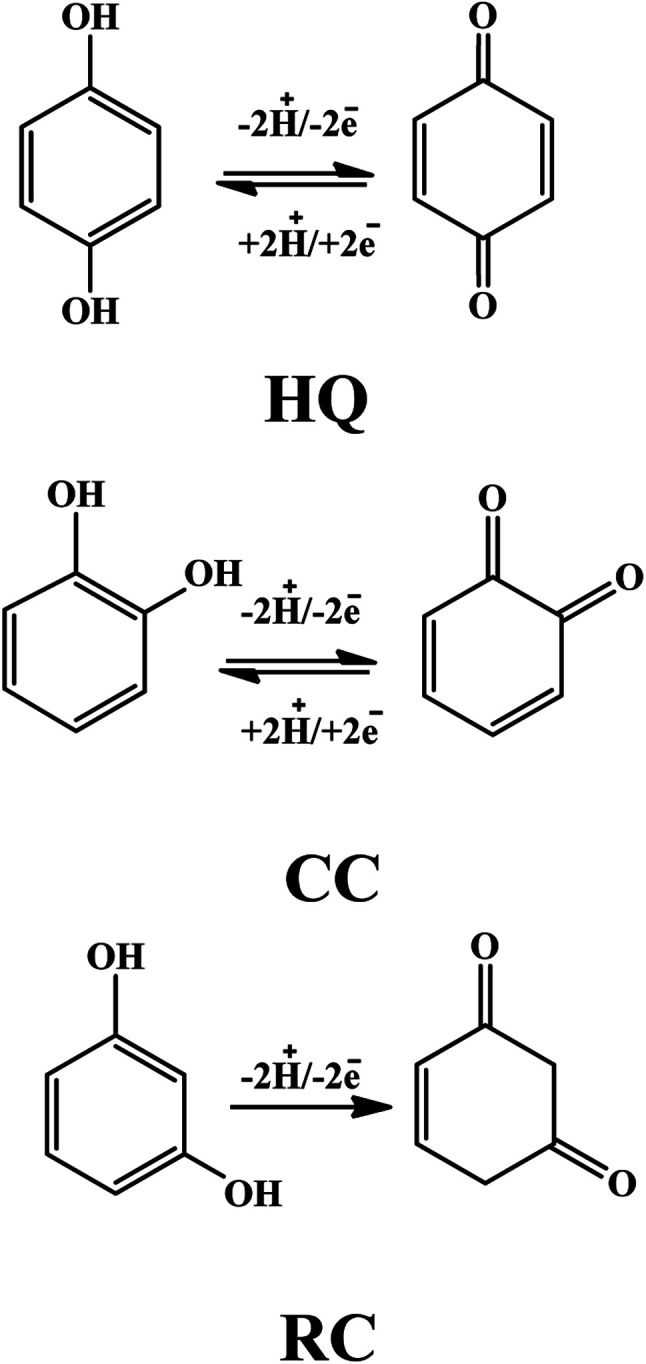
Proposed pH dependent redox reactions of HQ, CC, and RC.

The pH value can fluctuate the position and current amplitude of the signals. Variation in peak position suggests the involvement of protons during electrooxidation of HQ, CC, and RC at the electrode surface. For the DHBIs, the peak currents variations with the increase of pH from 3.0 to 7.6 are shown in Fig. 4B. The gradual increase of DHBIs signals till pH 6 indicates the existence of isomers (p*K*a = 9.0) in acid–base equilibrium (Fig. 4C). At higher pH, the decrease in signals height can be attributed to the likely deprotonation of DHBIs and oxygen functionalities of fCNTs. Consequently, the kinetically unfavourable oxidation reactions related to the electrostatic repulsion at the sensor/electrolyte interface may lead to unstable and inefficient adsorption of DHBIs in alkaline media. Thus, to achieve excellent sensitivity and well resolved DHBIs peaks at fCNTs/Au–Ag NPs/fCNTs/GCE, the PBS of pH 6 was used. The linear plots of *E*_p_*vs.* pH are nearly parallel showing a steady peak to peak difference in the studied pH range as illustrated in Fig. 4D. Moreover, the slope of *E*_p_*vs.* pH plots (Nernstian slope 58.5 mV pH^−1^)^1^ suggests the involvement of equal number of protons and electrons in the redox reaction of DHBIs at the nanosensor surface. The proposed pH dependent electro-oxidation mechanism of DHBIs can be seen in Scheme 1.

**Scheme 2:**
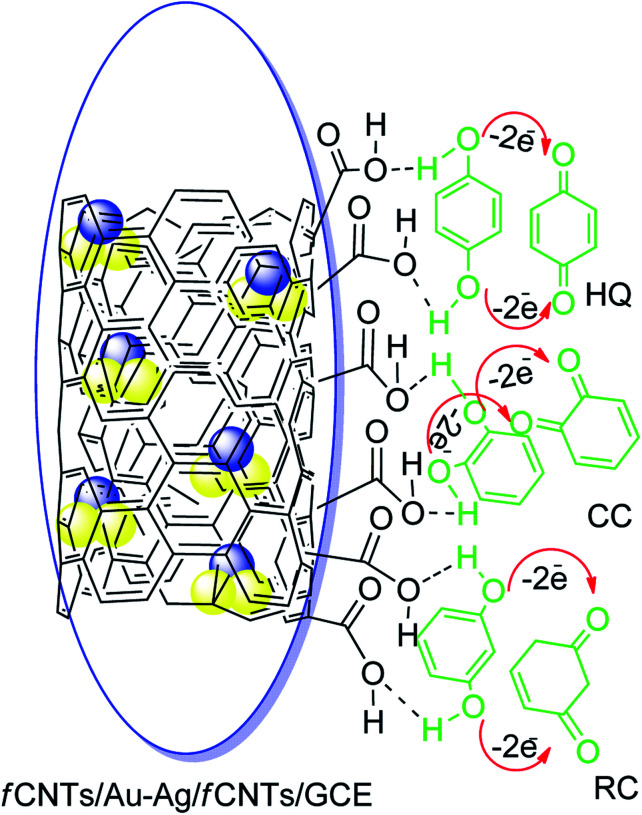
Suggested oxidation mechanism of HQ, CC, and RC at the designed nanosensor.

The superior performance of the designed nanosensor for the electrooxidation of HQ, RC, and CC can be credited to the synergistic role of fCNTs and Au–Ag NPs (Scheme 2).^38^ The fCNTs facilitate electron transfer rate due to their conductive nature and support adsorption of DHBIs during the pre-concentration step of SWASV.^39–41^ Likewise, the fCNTs have different space resistances for different DHBIs, thereby improving the voltammetric discrimination between the isomers by lowering their overpotentials.^16,41^ Moreover, the oxygen functionalities of the fCNTs can develop hydrogen bonding with the DHBIs hydroxyl groups (H⋯O–H).^27,42^ In addition, the hydrogen bonds may also be developed between the surface adsorbed analytes and their aqueous dissolved species.^43^ The rings of CNTs can offer additional π–π interactions to the benzene unit of DHBIs *via* solute–sorbent interactions to boost their regional concentration and enhance electrocatalytic redox events (Scheme 2). Moreover, metal NPs exert their electrocatalytic role for the oxidation of the DHBIs *via* metal nuclei active sites and the mechanism follows conventional heterogeneous catalysis process as reported by the previous investigators.^25,43^ The combined effect of all the above mentioned interactions giving rise to a sensitive electrochemical scaffold for the discrimination and concurrent sensing of all the three isomers has been portrayed in Scheme 2.

### 3.6. Analytical features of the designed nanosensor

**Fig. 5:**
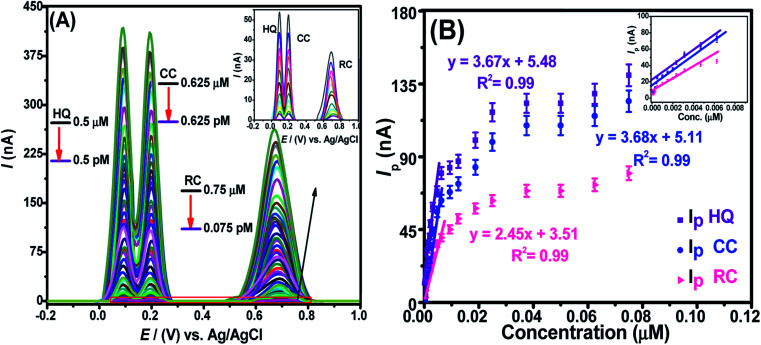
(A) The SWASV noted by synchronizing conc. variation of HQ, CC, and RC in PBS of pH 6, keeping sweep rate of 100 mV s^−1^, deposition potential of 0 V, and accumulation time of 200 s. (B) Calibration plot of voltammetric data of DHBIs presenting linearity segments of concentrations with *I*_p_ values along with their regression equations under optimized conditions of fCNTs/Au–Ag NPs/fCNTs/GCE.

The detection limit (LOD) and the quantification limit (LOQ) can be evaluated from their typical descriptions (LOD = 3*σ*/*m* and LOQ = 10*σ*/*m*), where “*σ*” signifies the standard deviation of “*n*” times repeat of voltammograms of blank experiments (in electrolyte solution only) performed at the nanosensor and calibration plot (conc. *vs. I*_p_) slope “*m*”. Likewise, the linear concentration ranges (LCRs) relate to the liner segment of the calibration plot. We assessed these parameters for DHBIs under optimized conditions at fCNTs/Au–Ag NPs/fCNTs/GCE by SWASV. The results show a steady rise in *I*_p_ of HQ, RC, and CC with continuous addition of their investigated concentrations (Fig. 5A). The corresponding calibration curves shown in Fig. 5B were used for the evaluation of the sensor's performance parameters as listed in Table S2.† The femtomolar LODs of the target analytes demonstrates the superior figures of merit and selectivity of the designed nanosensor. The trend of sensitivity at the sensing scaffold follows the sequence; HQ > CC > RC which is in accordance with the electro-activities of these isomers.

### 3.7. Reproducibility, reusability, and stability of the designed sensor

The accuracy and precision of the nanosensor was examined by evaluating its reusability, reproducibility, and stability. For reproducibility test experiments were performed under the same conditions on six GCEs modified with the same nanocomposite (fCNTs/Au–Ag NPs/fCNTs) as illustrated in Fig. S5A,† while stability and reusability characteristics were investigated over a consistent time interval at the same nanosensor as revealed in Fig. S5B.† The fCNTs/Au–Ag NPs/fCNTs/GCE was reserved in a desiccator at RT when not in use during repeatability analysis. The current response of the tri-analytes DHBIs mixture under optimized conditions with variation of less than 2% RSD pointed to the promising reliability of the designed sensing scaffold.

An evaluation of the analytical features of the designed sensing platform with the already reported systems for the solo and concurrent detection of the HQ, CC, and RC is specified in Table 1. The calculated figures of merit especially the LOD values are far better than the suggested limits for these toxic hydrophilic isomers.^30^ Remarkably, the LODs for the target DHBIs attainable by using the stated platform are much better than any sensors reported so far.^2–6,8,10–15^ Hence, the designed electrochemical nanosensor is novel in the perspective of sensitivity, stability and selectivity.

**Table 1:** Assessment of the sensing performance of fCNTs/Au–Ag NPs/fCNTs with reported sensors for HQ, CC, and RC

Sensors	Measurement technique	LOD (nM)			Ref.
		HQ	CC	RC	
HMCCSs^a^/GCE	DPV	120	190	1100	2
Au/lys/OMC-Au/Tyr/GCE	DPV	50	25	N.M	3
SiO_2_/C/Nb_2_O_5_	DPV	1200	800	N.M	4
*p*-DMcT/GCE	DPV	100	100	300	5
Co-SnO_2_ NPs	SWV	450	94	N.M	6
DAT-COF/GCE		130	70	80	8
NH_2_-SBA 15-silica/CPE	DPV	300	500	800	10
PDDA-G-GCE	DPV	250	200	N.M	11
NCNF/GCE	DPV	300	400	800	12
P-rGO/GCE	DPV	80	180	2620	13
NaOH/GCE	SWV	10	10	90	14
GSEC	SWV	100	100	50	15
**fCNTs/Au–Ag NPs/fCNTs/GCE**	**SWASV**	**28.6 fM**	**36.5 fM**	**42.8 fM**	**This work**

a HMCCSs = hollow molybdenum-dopamine spheres; lys = lysine; Tyr = tyrosinase; Au NPs = gold nanoparticles; *p*-DMcT = 2,5-dimercapto-1,3,4-thiadiazole; DAT-COF = 3,5-diamino-1,2,4-triazole-CO covalent organic framework film; NH_2_-SBA 15-silica/CPE = amino functionalized ordered mesoporous silica/carbon paste electrode; PDDA-G = poly(diallyldimethylammonium chloride) functionalized graphene; NCNFs = nitrogen-doped carbon nanofibers; P-rGO: porous graphene; GSEC = graphene sheets embedded carbon.

b N.M = not measured.

### 3.8. Interference study for practical applicability of the sensor

For examining the effect of interfering agents on the sensing performance of the designed nanosensor, the electrochemical responses of the DHBIs were equated in the absence and presence of the interferents under optimized conditions. The voltammograms and bar plots of the voltammetric response of 0.5 μM HQ, 0.625 μM CC, and 0.75 μM RC mixture in the coexistence of 2 mM amount of each organic/inorganic interferents (details given in Fig. S6† caption) can be seen in Fig. S6A & B.† The tolerance level of the designed sensor was observed as a % RSD <5% for these interferents. These outcomes manifest the exactness of the suggested sensor for the target analytes in the presence of multifold higher amounts of the interferents.

### 3.9. Investigation of real samples

For validity of the fCNTs/Au–Ag NPs/fCNTs/GCE in water resources, HQ, CC, and RC were simultaneously analysed in 3-matrixes of each real sample (drinking, tap, spring, rain, lake, river, sea, and artificial wastewater, spinach, and onion juices). First, initial amounts of the DHBIs were assessed in all samples by SWASV and then known amounts of the isomers were spiked into the real samples and % recoveries were determined. The signal values of DHBIs in real samples were matched with their calibration plots shown in Fig. 4B. Each reading was triplicated and % recoveries in the range of 96% to 105% with % RSD <2.5% were attained as summarized in Table S3.† The data validates the fCNTs/Au–Ag NPs/fCNTs/GCE for workable environmental analysis.

### 3.10. Computational studies

**Fig. 6:**
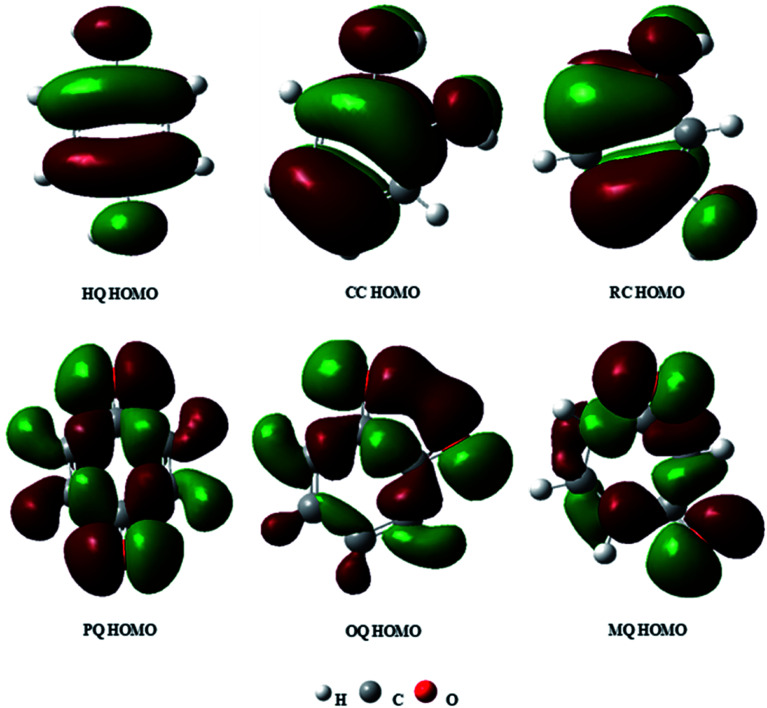
Pictorial picture of HOMO of optimized structures of HQ, CC, RC, PQ, OQ and MQ by DFT on Gaussian 09 software B3YLP/6-311G + + (d, p) in water solvent.

For the perception of a molecular level detection of DHBIs and their respective quinones at the designed sensor, the quantum parameters of the cited toxins were studied theoretically (see Table S4†) to compare their qualitative trends as witnessed in voltammetric analysis. The HOMO (highest occupied molecular orbital) of the HQ, CC, and RC optimized structures and their respective ortho- para- and meta-quinones (Scheme 1) are portrayed in Fig. 6. The chemical reactivity of DHBIs related to their bandgap energy (*E*_g_) shows minimum *E*_g_ of HQ (0.191 Hartree) which can be ascribed to its ease electrochemical oxidation than CC (0.199 Hartree) and RC (0.213 Hartree) (see Fig. 3). Likewise, other chemical descriptors of DHBIs listed in Table S4† also verify the experimental observation that HQ oxidizes before CC and RC and hence easily detectable owing to its kinetically more polarizable molecular structure.^32^ Conversely RC is difficult to oxidize (as witnessed by its peak potential value in CV and SWAS voltammograms shown in Fig. 3) owing to its highest ionization energy (IE). The higher electron affinity (EA) and lower IE values of HQ and CC indicate the formation of thermodynamically stable oxidized products, as witnessed by the reversibility of HQ and CC in CV profiles (Fig. 3A). On the contrary, lower EA and higher *E*_LUMO_ value of RC infer release of small amount of energy on the reduction of meta-quinone (quinone formed from RC oxidation) at CV reduction scan, which disfavours the reduction of RC to MQ due to instability of the product.^4^ Meanwhile, two carbons convert their hybridization state from sp^2^ to sp^3^ to generate MQ which is thermodynamically unstable, accordingly no reversibility of RC is observed in cyclic voltammograms (Fig. 3A). Thus, these analyzed theoretical values strongly support the qualitative electrochemical aspects of the DHBIs at the designed nanosensor.

**Fig. 7:**
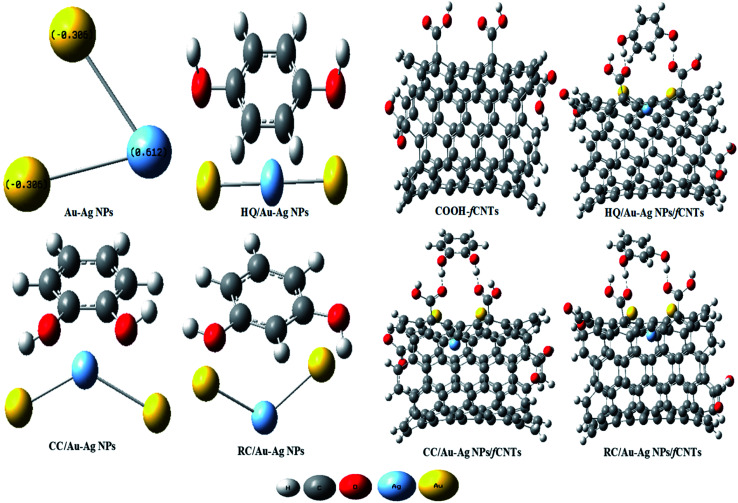
The optimized structures of Au–Ag NPs (single atoms of Au and Ag), fCNTs (4-COOH group on single unit of armchair (5, 5)) and their merged systems with HQ, CC, and RC by M06-2X method on Gaussian 09 software in water solvent.

The key steps included in the sensing mechanism at the modified electrode involve adsorption of DHBIs molecules at the sensor surface and the synergistic role of the components of nanocomposite in facilitating charge transfer host (transducer) and guest (analyte). The catalytic mechanism at the designed sensor can be proposed on the basis of quantum descriptors of the DHBIs/Au–Ag NPs and DHBIs/fCNTs/Au–Ag NPs (Fig. 7 & Table S5†). The negative binding energies (Δ*E*(HQ) −0.15 Hartree, Δ*E*(CC) −0.09 Hartree and Δ*E*(RC) −0.03 Hartree) of DHBIs at the Au–Ag NPs surface indicate activation of the O–H functionalities of the DHBIs on interaction with Milliken charges of the Au–Ag NPs.^32^ Thereby, elongation of O–H bond, increase of polarizability (*α*) and reduction in energy gap (*E*_g_) of DHBIs at Au–Ag NPs lead to their easier oxidation than free molecules. The calculated quantum properties listed in Table S5† reveals more stable adsorption and facile oxidation of HQ than CC and RC at Au–Ag NPs.

The negative Δ*E* values of the COOH-fCNTs/Au–Ag NPs/DHBIs merged system (Δ*E*(HQ) −5.6 Hartree, Δ*E*(CC) −4.1 Hartree, and Δ*E*(RC) −2.3 Hartree) than DHBIs/Au–Ag NPs suggest DHBIs capturing ability of the sensor owing to the synergy of Au–Ag NPs and COOH-fCNTs (Fig. 6, Table 2). Likewise, the decrease in *E*_g_ values at sensor surface corresponds to polarizable DHBIs molecules that further support the stable adsorption of DHBIs at the nanocomposite surface *via* non-covalent interactions (Fig. 3).^33^ The trend of HQ > CC > RC inferred from Δ*E* and reactivity quantities of the DHBIs at Au–Ag NPs/COOH-fCNTs is in accordance with the LOD values of DHBIs (Table S2†). These theoretical findings not only endorse the authenticity of the experimental conclusions, but also verify the role of fCNTs/Au–Ag NPs/fCNTs as a facilitator of charge transport at the electrode surface through non-covalent host (GCE)-guest (DHBIs) interactions.

**Table 2:** The electronic properties of fCNTs (4-COOH groups on single armchair (5, 5)) and their DHBIs/Au–Ag NPs/fCNTs merged systems (Hartree units) calculated by function M06-2X with 6-311G + + (d, p) and LANL2DZ basis sets, respectively in water solvent

Structural parameters	*E*	*E*_HOMO_	*E*_LUMO_	*E*_g_	IE	EA	Δ*E*
fCNTs	−4198.38	−0.154	−0.101	0.053	0.154	0.101	
HQ/Au–Ag NPs/fCNTs	−4992.36	−0.127	−0.092	0.035	0.127	0.092	−5.6
CC/Au–Ag NPs/fCNTs	−4993.72	−0.153	−0.111	0.042	0.153	0.111	−4.1
RC/Au–Ag NPs/fCNTs	−4995.44	−0.171	−0.123	0.048	0.171	0.123	−2.3

## 4. Conclusion

Results of our experiments demonstrate that GCE modified with fCNTs/Au–Ag NPs/fCNTs is an efficient platform for the concurrent ultrasensitive detection of dihydroxybenzene isomers (DHBIs) *i.e.*, hydroquinone (HQ), resorcinol (RC) and catechol (CC) isomers. Compared to bare GCE the designed sensing platform shows well resolved signals with amplified peak currents and considerable lowering of over potentials. The improved performance is credited to the greater active surface area and conductivity of the modified electrode surface. The designed senor shows wide LCRs and femtomolar LOD values of DHBIs as compared to any reported sensor for the isomeric phenolic water toxins. The designed sensor also exhibits antifouling properties, stability and reproducibility. Moreover high percentage recoveries in a number of real water samples indicate practical applicability of the sensor for on-site simultaneous detection of the target isomers. DFT results suggest the existence of non-covalent interactions between the Au–Ag NPs/fCNTs and DHBIs that result in improved electrocatalytic performance of the modified electrode surface.

## Conflicts of interest

There are no conflicts to declare.

## Supplementary Material

RA-011-D1RA05669G-s001
