# Supplementary material for: Phenolic water toxins: redox mechanism and method of their detection in water and wastewater
Source: RSC Adv. 2021 Nov 5;11(57):35783–95. doi: 10.1039/d1ra05669g (PMC9043152; doi:10.1039/d1ra05669g)
Supplement: RA-011-D1RA05669G-s001 [file RA-011-D1RA05669G-s001.pdf]

Electronic Supplementary Material (ESI) for RSC Advances.
This journal is © The Royal Society of Chemistry 2021

Supporting Information

Phenolic Water Toxins: Redox Mechanism and Method of Their Detection in Water and Wastewater

Tayyaba Kokab [a], Afzal Shah [a, *], Jan Nisar [b], Muhammad Naeem Ashiq [c], M. Abdullah Khan [d] , Sher Bahadar Khan [e] and Esraa M. Bakhsh [e]

[a]Department of Chemistry, Quaid-i-Azam University, Islamabad, 45320, Pakistan

[b]National Centre of Excellence in Physical Chemistry, University of Peshawar, Peshawar 25120, Pakistan

[c]Institute of Chemical Sciences, Bahauddin Zakaryia University, Multan 6100, Pakistan

[d]Renewable Energy Advancement Laboratory, Department of Environmental Sciences, Quaid-i-Azam University, Islamabad, 45320, Pakistan

[e]Department of Chemistry, King Abdulaziz University, P.O. Box 80203, Jeddah 21589, Saudi Arabia

*To whom correspondence should be addressed

E-mail: Dr. Afzal Shah (afzals_qau@yahoo.com)

1. Synthesis of electrode modifier and its use for modification of GCE

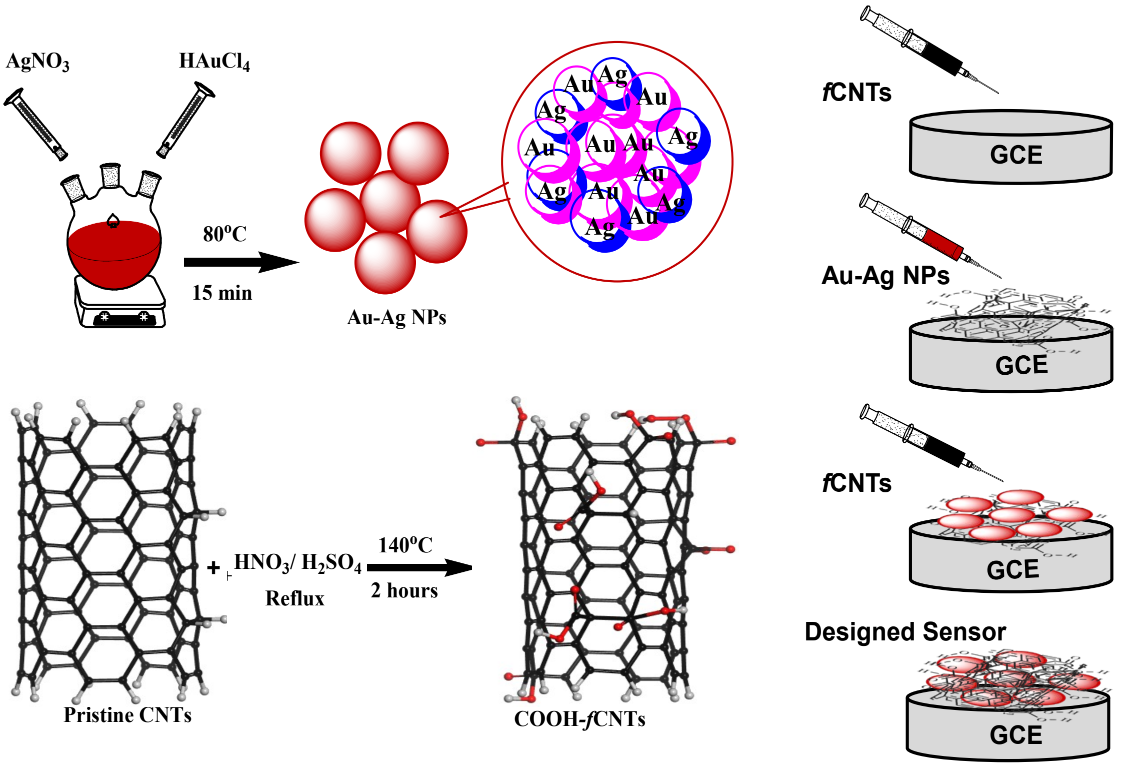

Scheme S1. The development process of *f*CNTs/Au-Ag NPs/*f*CNTs nanocomposite based sensor.

2. EIS and CV Experimental Data

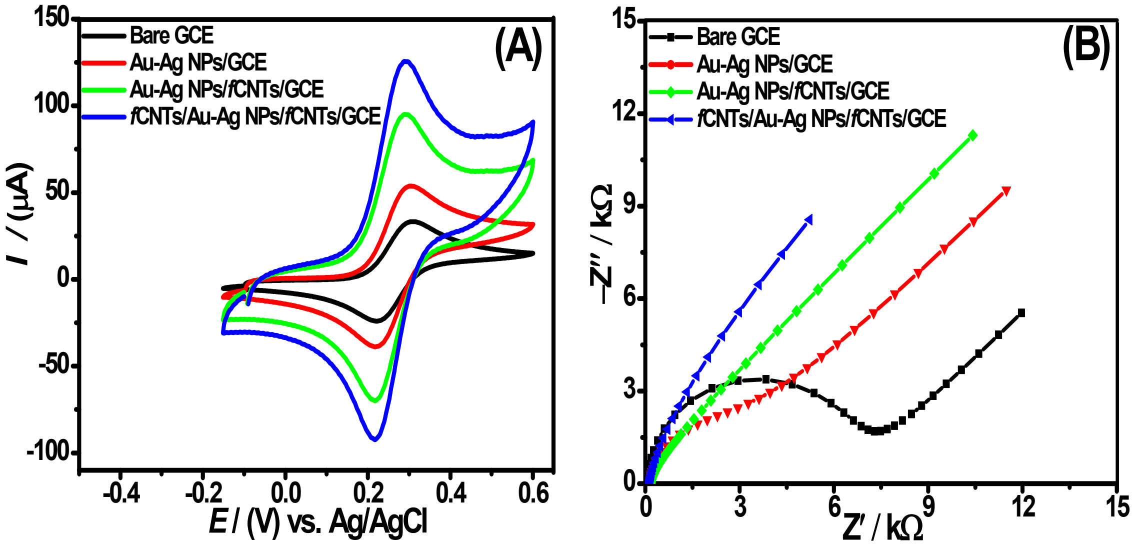

Figure S1. (A) Cyclic voltammograms of 5 mM $K_3[Fe(CN)_6]$ with 0.1 M KCl electrolyte at modified and unmodified GCE at a scan rate 100 mV/s. (B) Nyquist plots of 5 mM $K_3[Fe(CN)_6]$ with 0.1 M KCl electrolyte using data obtained at bare GCE and modified GCEs.

Table S1 The Randles equivalent circuit model fitting with the EIS parameters, and CV: calculated data for bare and modified GC electrodes.

**Electrodes**	**Bare GCE**	**Au-Ag NPs/GCE**	**Au-Ag NPs/ *f*CNTs/GCE**	***f*CNTs/Au-Ag NPs/ *f*CNTs/GCE**
**Area (cm^2)**	0.02	0.04	0.07	0.11
**R_{ct} (Ω)**	6450	2642	2.3×10^{-5}	1.5×10^{5}
**Re (Ω)**	83.53	153	141.9	111.2
**CPE (μF)**	0.71	2.01	5.67	29.2
**n**	0.69	0.83	0.9	0.97

3. Optimization of voltammetric experimental parameters

Prior to the proposed SWASVs method's application, experimental conditions were optimized for the best performance of the designed sensor.

3.1. The influence of the amount of modifier

The amount of *f*CNTs and Au-Ag NPs, and the order of their application at the electrode surface influence the response of DHBIs. In this regard, single nanostructures (Au-Ag NPs, CNTs and *f*CNTs), different blends of Au-Ag NPs and *f*CNTs, layer by layer (LBL) and dispersion strategies were adopted for the sensor development. The best sensing results were achieved by adopting LBL fabrication process for the preparation of *f*CNTs/Au-Ag NPs/ *f*CNTs/GCE sandwiched nanosensor. Thus, various amounts of *f*CNTs and Au-Ag NPs were applied on GCE by drop casting LBL strategy. The SWASV was run to inspect the amount of the nanostructures for attaining the maximum current signals of the target isomers while other detecting conditions were kept invariant. The relationship between isomers peak currents and drop casted amounts of modifiers immobilized on the GCE surface can be seen in **Figure S2**. The increase of modifier amount increases the current response, while intense current signals were obtained with 4µL *f*CNTs, 1µL Au-Ag NPs and 4µL *f*CNTs. On further increase of modifiers amount, the oxidation signals were reduced in intensity possibly due to over thick layer deposition leading to electrical resistance and instability of the modified electrode. Thus, a too thick or too thin layer of nanostructures showed adverse behaviour for the electrochemical response. Hence, an optimized ratio 4µL: 1µL: 4µL of *f*CNTs: Au-Ag NPs: *f*CNTs modified electrode was used to perform further experiments.

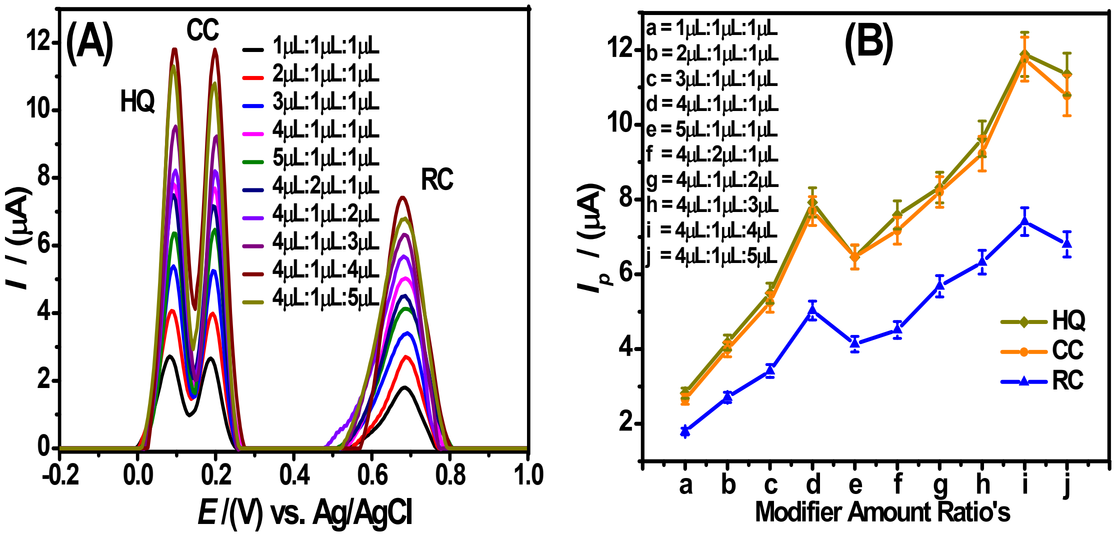

Figure S2. (A) Modifiers amount effect on the SWASVs response of HQ (10 μM), CC (12.5 μM), and RC (15 μM) mixture in pH 6 PBS as striping solvent, at sweep rate 100 mV/s, deposition potential 0 V and accumulation time of 5 s through LBL modification of GCE surface from different ratios of *f*CNTs: Au-Ag NPs: *f*CNTs respectively (B) Plot of I_p of the isomers mixture vs modifiers *f*CNTs: Au-Ag NPs: *f*CNTs ratios with error bars.

3.2. Deposition potential and deposition time

The adsorption quantity is primarily controlled by the properties of the analyte molecules and the electrode surface state. The substrate molecules first transport from the solution in the electrochemical cell to the GCE surface and get immobilized by the application of deposition potential for a certain accumulation time. The SWASVs preconcentration step was on HQ (10 μM), CC (12.5 μM) and RC (15 μM) mixture under open-circuit and magnetic stirring conditions to avoid mass transfer effect. The assemblage of HQ, CC and RC and electrochemical signals amplification was found to increase with rise in deposition potential and accumulation time. **Figure S3A & B** shows the effects of deposition potential on the magnitude of the current signals as a result of substrate immobilization on sensor surface from 0.3 V to 0.5 V. The highest current response for all the three isomers

were achieved at 0 V deposition potential. Furthermore, when the accumulation time increased from 5 s to 210 s at applied potentials of 0 V, the oxidation current intensified with increase of deposition time. The signals reached to their maximum height at 200 s and then the peak current-deposition time plot showed a decrease beyond 200 s (**Figure S3C & D**) due to saturation of electrode active sites. Therefore, 200 s accumulation time at 0 V was considered as optimized time for analytes deposition on the designed sensor surface.

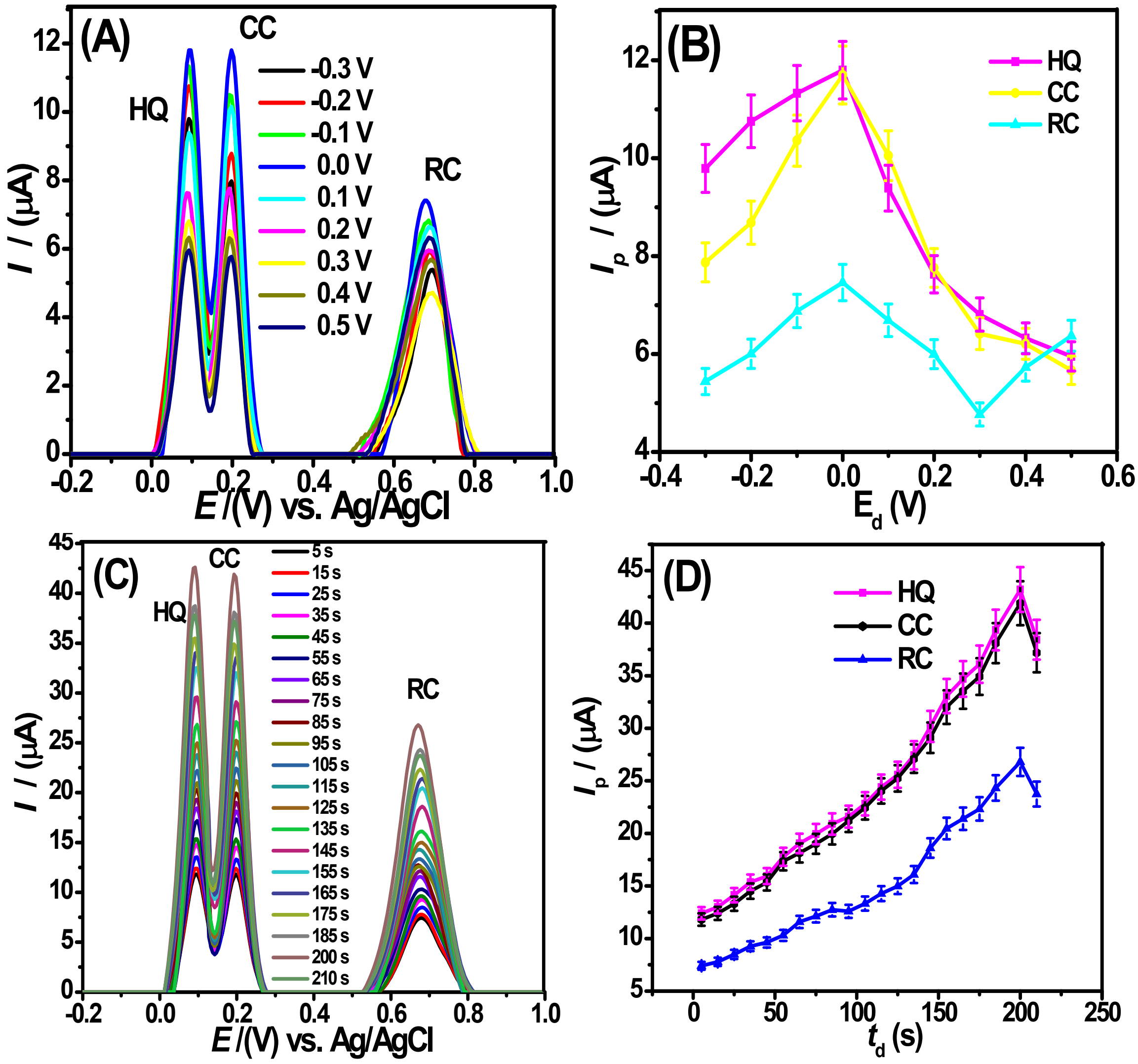

Figure S3 (A) Influence of accumulation potential on the SWASV current peak intensity of HQ (10 μM), CC (12.5 μM), and RC (15 μM) mixture in pH 6 PBS, at scan rate 100 mV/s, and accumulation time of 5 s through LBL modification of GCE with 4 μL *f*CNTs/1 μL Au-Ag NPs/4 μL *f*CNTs. (B) Plot of corresponding I_p vs E_d with error bar. (C) The deposition

time variation effect on the stripping current response of HQ (10 μM), CC (12.5 μM), and RC (15 μM) mixture at deposition potential of 0 V keeping other conditions invariant. (D) Plot between I_p vs t_d with error bar.

3.3. Effect of scan rate

The relationships of scan rate (υ) with the peak potentials (E_p) and peak currents (I_p) of the DHBIs were probed at *f*CNTs/Au-Ag NPs/*f*CNTs/GCE by varying the scan rate from 5 mV/s to 225 mV/s as illustrated in **Figure S4A**. The voltammograms show progressive increase of peak currents with increase of scan rate as expected. The direct relation of I_p and υ (R^2= 0.99) shown in **Figure S4B & C** signifies the likelihood of the adsorption-controlled process. Moreover, the slope of the log I_{pa} vs. log υ with value greater than 0.5 certifies the occurrence of surface-controlled process (**Figure S4D**). Though the peak potentials of HQ and CC shifted with the increase in υ, yet the potential difference (ΔE_p=E_{pc} - E_{pa} ~ 30 mV) remained constant. The assessment of ΔE_p value with 59/n mV reveals that two electrons are involved in the electrochemical redox processes of the HQ and CC (**Scheme S2**).[1] Besides the ratio of I_{pa}/I_{pc} with a value close to 1 corroborates with the reversible nature of the redox reaction of HQ and CC at the sensor surface. For RC, the positive shift in peak potential with the υ is a representative feature of the irreversible oxidation process. The slope value (0.064 V) of the linear plot of E_p vs. log υ according to the Laviron equation suggests that that oxidation of RC is a two-electron transfer process (**Scheme S2**). Thus, HQ, CC, and RC undergo two electrons oxidation processes at the nanosensor surface.

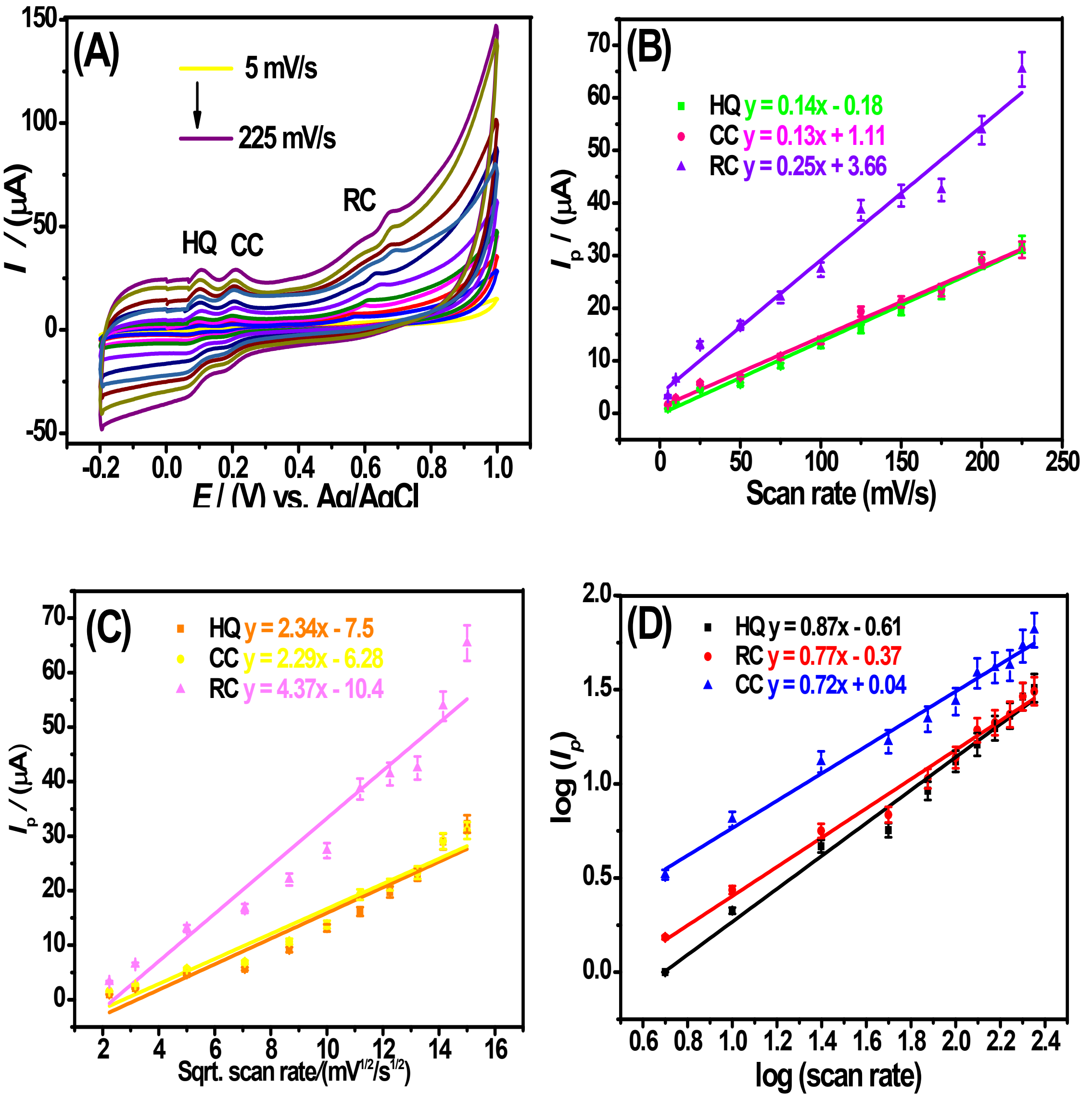

Figure S4 (A) Cyclic voltammograms of HQ (12.5 μM), CC (15 μM), and RC (17.5 μM) mixture at *f*CNTs/Au-Ag NPs/*f*CNTs/GCE in PBS of pH 6 with scan rate varying from 5 mV/s to 225 mV/s in the potential domain of 0.2 V to 1.0 V (B) Plot of peak currents of HQ, CC and RC vs scan rate (mV/s) (C) I_p plot vs square root of scan rate (D) Plot of log of I_p vs log scan rate.

4. Figures of merit of the designed sensor

Table S2 Figures of merit of *f*CNTs/Au-Ag NPs/*f*CNTs/GCE for the detection of HQ, CC, and RC.

Figures of Merits	Units	HQ	CC	RC
Investigated range	µM to pM	0.5 — 0.5	0.625 — 0.625	0.75 — 0.75
Linearity range	nM to pM	6.25 — 0.5	7.8 — 0.625	9.37 — 0.75
LOD	fM	28.6	36.5	42.8
LOQ	fM	95.3	121.6	142.6
% RSD (Reproducibility)	n=6	0.6	1.26	1.59
% RSD (Repeatability)	n=5	0.35	0.58	0.4
% RSD (Stability)	n=8	1.14	1.92	2.21
% RSD (Anti interference ability)	n=20	0.872	1.32	3.43
% RSD (Validity)	n=6	< 2.5	< 2	< 2.5
% Recovery	n=13	96 —105	97 —104	97 — 104

5. Reproducibility, reusability, and stability of the designed sensor

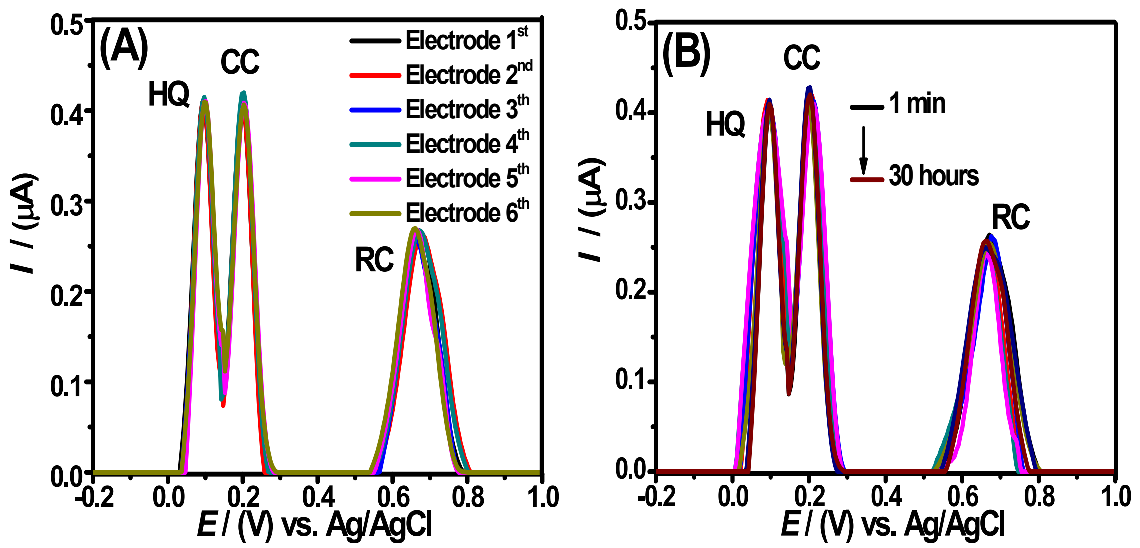

Figure S5 Validity of the proposed procedure authenticated by inspecting the SWASV peak currents I_p of HQ (0.5 µM), CC (0.625 µM) and RC (0.75 µM) under predefined optimized conditions showing (A) reproducibility of multiple fabricated *f*CNTs/Au-Ag NPs/*f*CNTs/GCEs (n=6), (B) repeatability, reusability and stability of the *f*CNTs/Au-Ag NPs/*f*CNTs/GCE at intra and inter days scans.

6. Interference study for validation of sensor

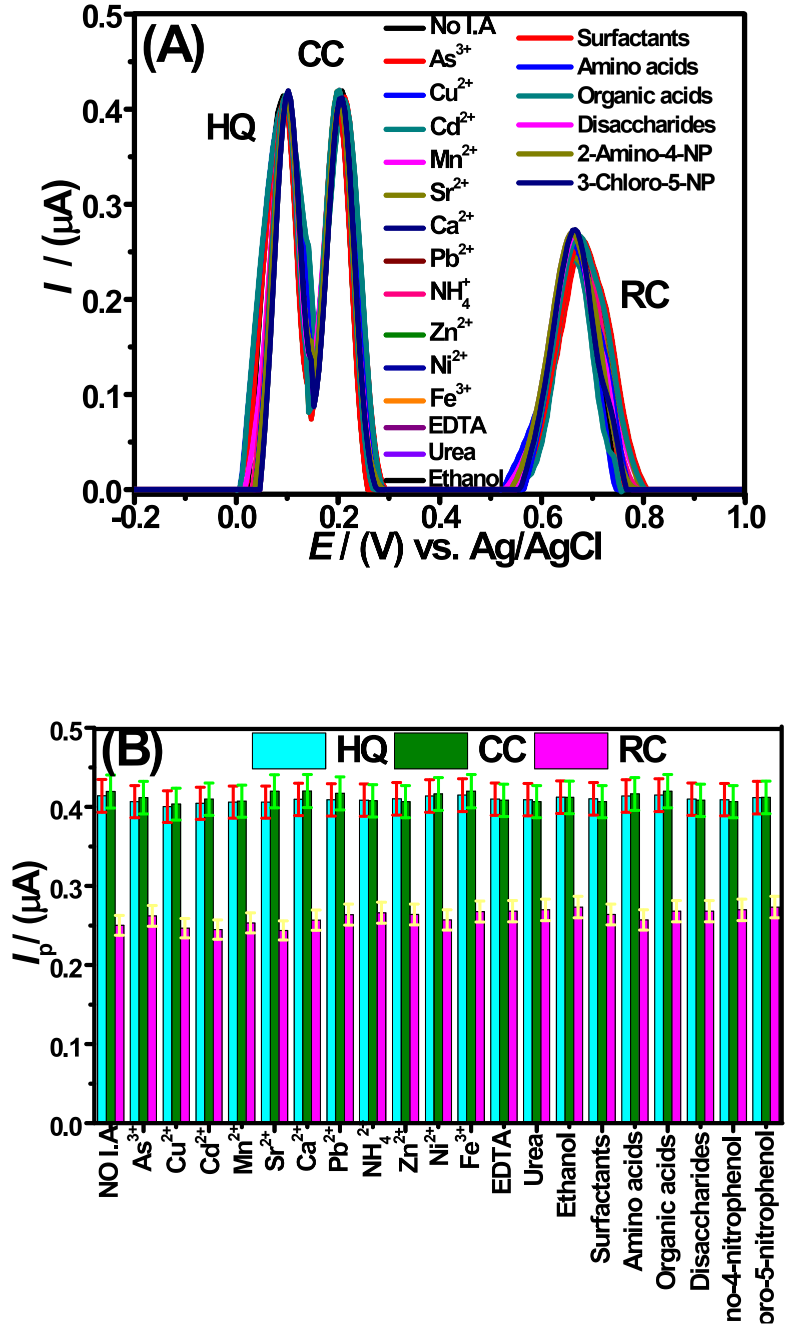

Figure S6 (A) Voltammograms of the DHBIs achieved with *f*CNTs/Au-Ag NPs/*f*CNTs/GCE in the presence of 2 mM of one of the interferents i.e. metal ions (As^{3+}, Cu^{2+}, Cd^{2+}, Mn^{2+}, Sr^{2+}, Ca^{2+}, Pb^{2+}, NH_4^+, Zn^{2+}, Ni^{2+}, Fe^{3+}), EDTA, Urea, Ethanol, Surfactants (SDS,CTAB), Amino acids (threonine, glycine, alanine, glutamic acid, cysteine), organic acids (tannic acid, fumic acid, citric acid, oxalic acid, salicylic acid), disaccharides (glucose, maltose, fructose,

sucrose, lactose), 2-amino-4-nitrophenol, 3-chloro-5-nitrophenol with HQ (0.5 μM), CC (0.625 μM), and RC (0.75 μM) mixture in PBS of pH = 6 under optimum conditions of SWASV. (B) Resultant bar graph with error bars.

7. Real sample analysis

Table S3. Detection results of hydroquinone, catechol, and resorcinol in real samples obtained under the optimized SWASV conditions.

Samples	Sr #	Initial Amounts			Spiked (nM)			Found (nM)			Recovery (%)		
		HQ	CC	RC	HQ	CC	RC	HQ	CC	RC	HQ	CC	RC
Drinking Water	1	-	-	-	2.5	2.5	2.5	2.55	2.5	2.4	102	100	96
	2	-	-	-	2.5	5	5	2.5	5.1	5.1	104	104	102
	3	-	-	-	5	6.5	7.5	5	6.4	7.5	100	98	100
Tap Water	1	-	-	-	2.5	2.5	2.5	2.5	2.45	2.5	100	98	100
	2	-	-	-	2.5	5	5	2.6	5.2	5.1	104	104	102
	3	-	-	-	5	6.5	7.5	5.1	6.6	7.6	102	101.5	101
Spring Water	1	-	-	-	2.5	2.5	2.5	2.5	2.55	2.45	100	102	98
	2	-	-	-	2.5	5	5	2.5	5.1	4.9	100	102	98
	3	-	-	-	5	6.5	7.5	5.1	6.4	7.4	102	98	98
Rain water	1	-	-	-	2.5	2.5	2.5	2.6	2.5	2.5	104	100	100
	2	-	-	-	2.5	5	5	2.6	5.1	5.15	104	102	103
	3	-	-	-	5	6.5	7.5	5.1	6.7	7.9	102	103	105

Lake Water	1	-	-	-	2.5	2.5	2.5	2.6	2.6	2.5	104	104	100
	2	-	-	-	2.5	5	5	2.6	4.9	5.2	104	98	104
	3	-	-	-	5	6.5	7.5	5.1	6.6	7.3	102	101.5	97
River Water	1	-	-	-	2.5	2.5	2.5	2.55	2.6	2.45	102	104	98
	2	-	-	-	2.5	5	5	2.6	5.1	4.95	104	102	99
	3	-	-	-	5	6.5	7.5	5.1	6.6	7.6	104	101.5	101
Sea Water	1	0.01	0.01	0.02	2.5	2.5	2.5	2.6	2.55	2.45	104	102	98
	2	0.02	0.01	0.02	2.5	5	5	2.55	5.1	4.9	102	102	98
	3	0.01	0.01	0.03	5	6.5	7.5	5.1	6.7	7.7	102	103	102
Artificial Waste-water	1	-	-	-	2.5	2.5	2.5	2.6	2.55	2.45	104	102	98
	2	-	-	-	2.5	5	5	2.6	5.2	5	104	104	100
	3	-	-	-	5	6.5	7.5	5.25	6.6	7.6	105	101.5	101
Spinach Juice	1	-	-	-	2.5	2.5	2.5	2.5	2.55	2.45	100	102	98
	2	-	-	-	2.5	5	5	2.55	5.2	4.95	102	104	99
	3	-	-	-	5	6.5	7.5	5.25	6.6	7.65	105	101.5	102
Onion Juice	1	-	-	-	2.5	2.5	2.5	2.6	2.55	2.4	104	102	96
	2	-	-	-	2.5	5	5	2.55	5.1	5	102	102	100
	3	-	-	-	5	6.5	7.5	5.2	6.6	7.3	104	101.5	97

8. Computational studies

8.1. Theoretical analysis of DHBIs

Table S4 Comparative data of DHBIs and their respective quinone quantum descriptors (in terms of Hartree units) calculated by DFT (B3LYP) method with 6-311G + + (d, p) basis set in water solvent.

Structural Parameters		HQ	CC	RC	PQ	OQ	MQ
**Total Energy (E)**		−383	−383	−383	−382	−382	−383
**Dipole Moment**		3.73	3.62	3.44	0.0	6.35	4.70
**E_{HOMO}**		−0.216	−0.222	−0.229	−0.287	−0.265	−0.247
**E_{LUMO}**		−0.025	−0.023	−0.016	−0.15	−0.15	−0.059
**Band Gap (E_g)**	[E_{LUMO} - E_{HOMO}]	0.191	0.199	0.213	0.14	0.12	0.19
**Ionization Energy**	IE (−E_{HOMO})	0.216	0.222	0.229	0.287	0.265	0.247
**Electron Affinity**	EA (−E_{LUMO})	0.025	0.023	0.016	0.15	0.15	0.059
**Electronegativity**	χ [(IE + EA)/2]	0.121	0.123	0.124	0.219	0.208	0.153
**Chemical Potential**	μ (−χ)	−0.121	−0.123	−0.124	−0.219	−0.208	−0.153
**Chemical Hardness**	η [(IE− EA)/2]	0.096	0.099	0.107	0.069	0.058	0.094
**Chemical Softness**	σ (1/η)	10.47	10.05	9.39	14.60	17.39	10.64
**Electrophilicity Index**	Ω ($\mu^2/2\eta$)	0.077	0.076	0.072	0.350	0.376	0.125

8.2. Theoretical analysis of designed sensor electrocatalytic mechanism

Table S5 Comparative data of DHBIs with their Au-Ag NPs merged systems quantum descriptors (in terms of Hartree units) calculated by M06-2X method with LANL2DZ basis set in water solvent.

**Structural Parameters**	**HQ**	**CC**	**RC**	**Au-Ag NPs**	**HQ/Au-Ag NPs**	**CC/Au-Ag NPs**	**RC/Au-Ag NPs**
**E**	−382.80	−382.72	−382.56	−416.75	−799.40	−799.38	−799.28
**ΔE**	-	-	-	-	0.15	0.09	0.03
**α**	76.53	76.67	76.89	113.57	215.89	217.62	220.62
**(O-H)**	0.96	0.96	0.96	-	0.99	0.98	0.99
**Bond length**	0.96	0.96	0.96		0.98	0.98	0.98
$\mathbf{E_{HOMO}}$	−0.217	−0.222	−0.229	−0.186	−0.134	−0.175	−0.209
$\mathbf{E_{LUMO}}$	−0.025	−0.023	−0.017	−0.127	−0.091	−0.093	−0.110
$\mathbf{E_g}$	0.191	0.199	0.212	0.059	0.043	0.082	0.099
**IE**	0.217	0.222	0.229	0.186	0.134	0.175	0.209
**EA**	0.025	0.023	0.017	0.127	0.091	0.093	0.110
**χ**	0.121	0.122	0.123	0.157	0.113	0.134	0.160
**μ**	−0.121	−0.122	−0.123	−0.157	−0.113	−0.134	−0.160
η	0.096	0.100	0.106	0.03	0.02	0.04	0.05
σ	10.47	10.03	9.42	33.9	46.51	24.39	20.20
**Ω**	0.077	0.075	0.071	0.415	0.294	0.219	0.257
